# Supplementary material for: Glucocorticoid Receptor Signaling in Myeloid Cells Orchestrates Inflammation Resolution and Muscle Repair
Source: Adv Sci (Weinh). 2026 Sep 13:e77653. Online ahead of print. doi: 10.1002/advs.77653 (PMC13572832; doi:10.1002/advs.77653)
Supplement: Supplementary file 1 [file ADVS-9999-e77653-s001.pdf]

Supplementary Information

Glucocorticoid Receptor Signaling in Myeloid Cells Orchestrates Inflammation Resolution and Muscle Repair

Sirine Souali-Crespo, Joe G. Rizk, Emilia Calvano, Rajesh Sahu, Emina Colovic, Imane Chabba, Valentine Gilbart, Erwan Grangirard, Bastien Morlet, Qingshuang Cai, Daniel Metzger, Delphine Duteil[#]

This file includes Supplementary Figures 1-4 and Supplementary Tables 1-3.

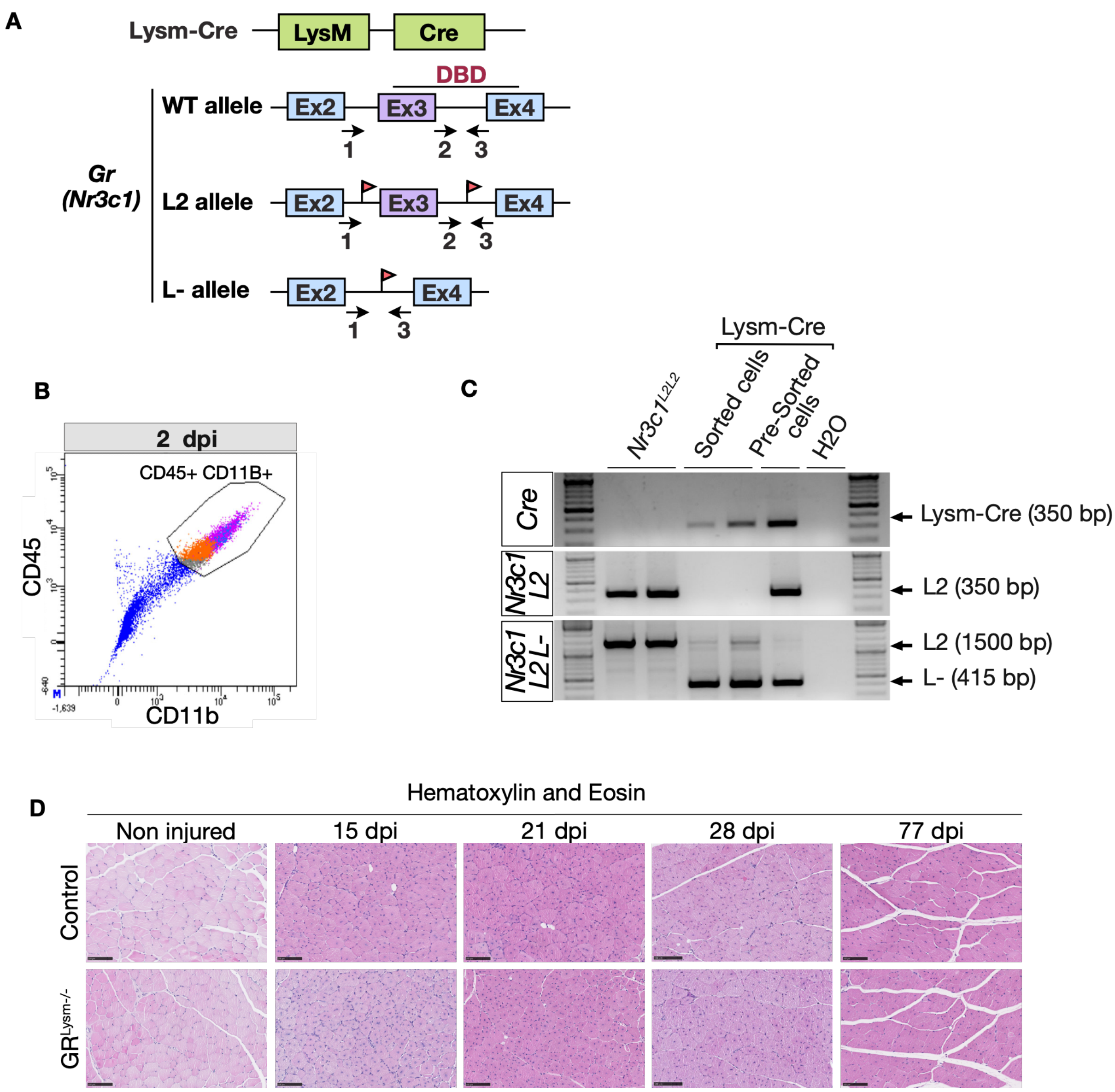

Figure S1: Efficient invalidation of GR in myeloid cells

(A) Schematic representation of *LysM*-Cre, wild-type (WT), floxed (L2) and Cre-mediated exon 3 deleted (L-) *Gr* alleles. LoxP sites are shown by arrowheads. Primers used for allele characterization are depicted by arrows and sequences are in material and method section.

(B) Representative flow cytometry density plot showing the gating strategy used to isolate $CD45^+/CD11b^+$ myeloid cells from injured TA muscles.

(C) Representative electrophoresis of PCR-amplified genomic DNA from control and $GR^{Lysm-/-}$ mice. Distinct amplicon sizes correspond to Cre (350 bp), GR L2 (1500 bp or 350 bp), and the recombined GR L– allele (415 bp). The absence of the GR L– band in $NR3C1^{L2/2}$ control mice confirms their genotype, whereas its presence in both FACS-sorted $CD45^+CD11b^+$ and presorted cell populations demonstrates successful recombination in the mutant.

(D) Representative hematoxylin and eosin staining of TA muscles of control and $GR^{Lysm-/-}$ mice under non-injured condition, and at 15-, 21-, 28- and 77-days post-injury (dpi). Scale bars, 100 µm.

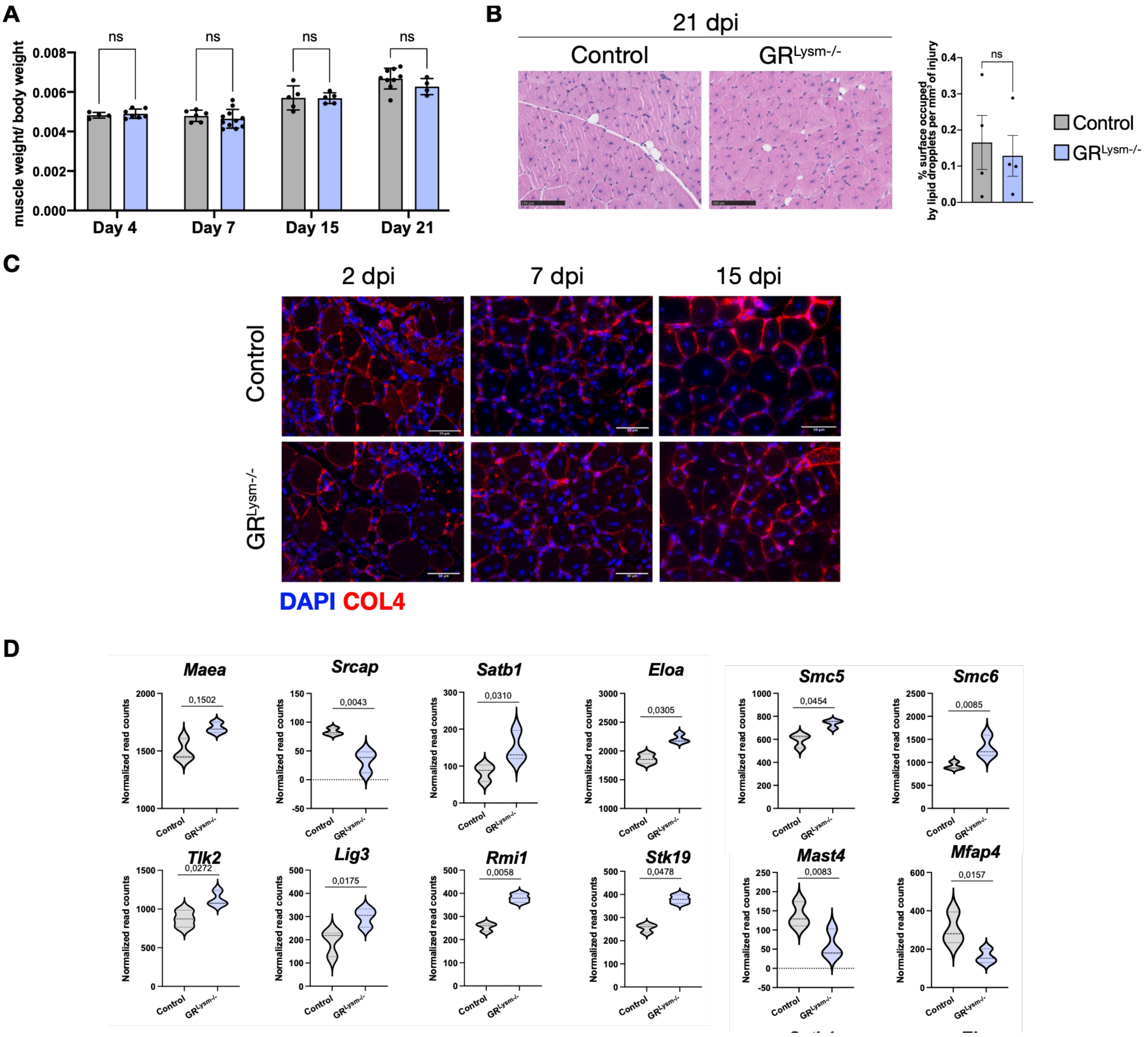

Figure S2: Effect of GR invalidation muscle homeostasis

(A) Muscle weight normalized to total body weight in injured mice at 4-, 7-, 15- and 21-days post-injury (dpi). Muscle mass was measured at the indicated time points following injury and normalized to each animal's total body weight. Day 4: Control mice: n=4; $GR^{Lysm-/-}$ mice: n=7. Day 7: Control mice: n=6; $GR^{Lysm-/-}$ mice: n=11. Day 15: Control mice: n=5; $GR^{Lysm-/-}$ mice: n=5. Day 21: Control mice: n=9; $GR^{Lysm-/-}$ mice: n=4. Data are presented as mean ± SEM. Statistical test used was Student's t-test; ns, non-significant.

(B) Representative hematoxylin and eosin staining of 21 days-injured TA muscles from control and $GR^{Lysm-/-}$ mice showing lipid droplet accumulation within the injury site. Quantification represents the surface area of lipid droplets relative to the total injury surface. Control mice: n=4; $GR^{Lysm-/-}$ mice: n=4. Data are presented as mean ± SEM. Statistical test used was Student's t-test; ns, non-significant.

(C) Representative immunofluorescent detection of COL4 (red) of TA of control and $GR^{Lysm-/-}$ mice at indicated time points of muscle regeneration. Scale bar, 15 µm.

(D) Violin plots showing normalized read counts of selected genes that were differentially expressed in bulk RNA-sequencing of FACS-sorted $CD11b^{+}$ cells isolated from TA muscles of control and $GR^{Lysm-/-}$ mice 7 dpi. Day 4: Control mice: n=3; $GR^{Lysm-/-}$ mice: n=3. *P*-values were obtained from DESeq2.

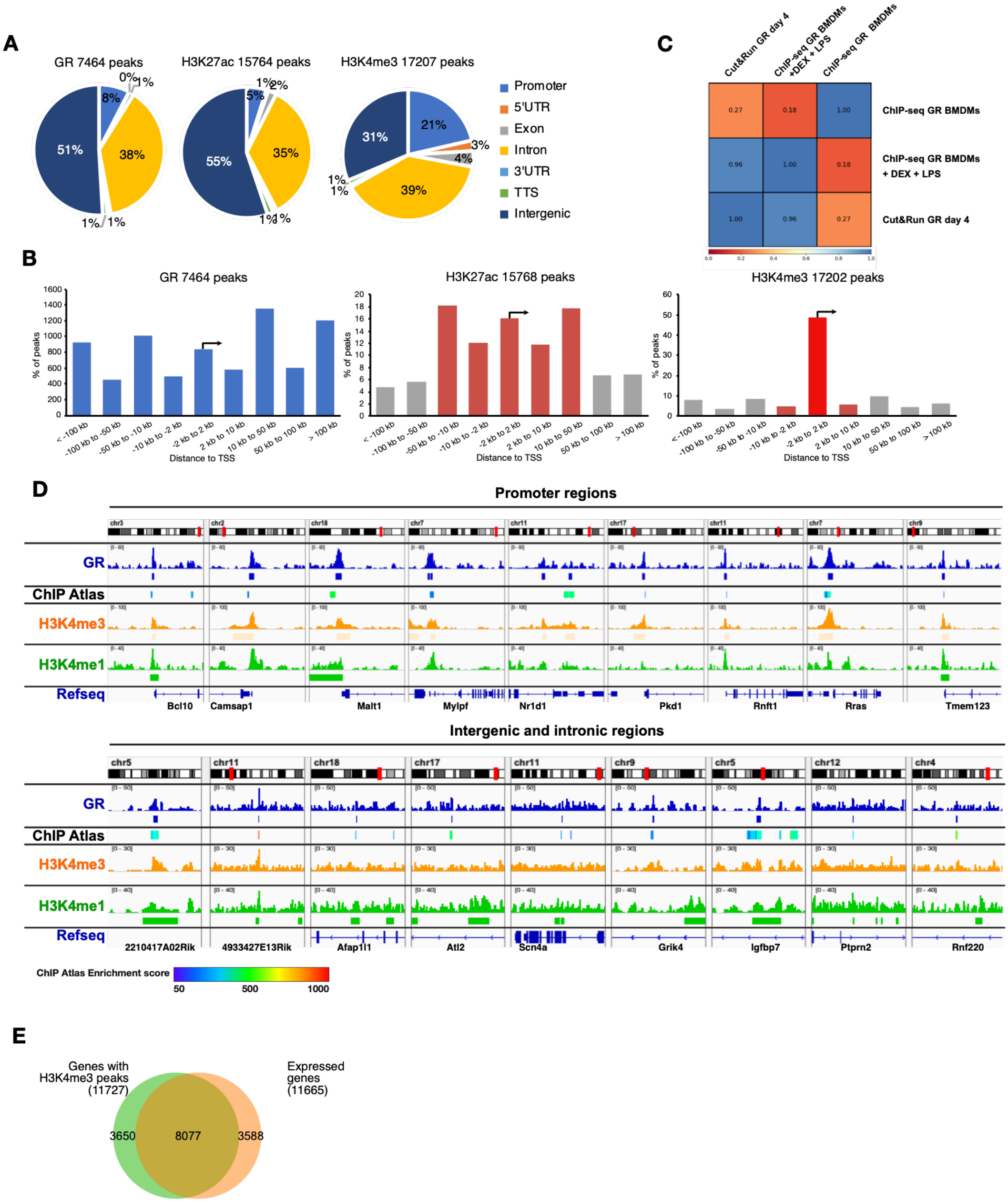

Figure S3: Integrative chromatin profiling of GR in myeloid cells isolated from TA muscle 4 dpi

(A) Pie chart depicting the genomic location of H3K4me3, H3K27ac and GR in macrophages.

(B) Distribution of H3K4me3, H3K27ac and GR peaks relative to their distance to the nearest TSS.

(C) Pearson correlation of GR in macrophages CUT&RUN analysis with public ChIP-seq datasets.

(D) Localization of GR, H3K4me3 and H3K27ac at representative promoter and intergenic regions on the chromatin of macrophages by CUT&RUN. Enrichment score showing the levels of confidence of published datasets is depicted as a heatmap.

(E) Overlap of the genes bound by H3K4me3 in macrophages expressed genes in myeloid cells.

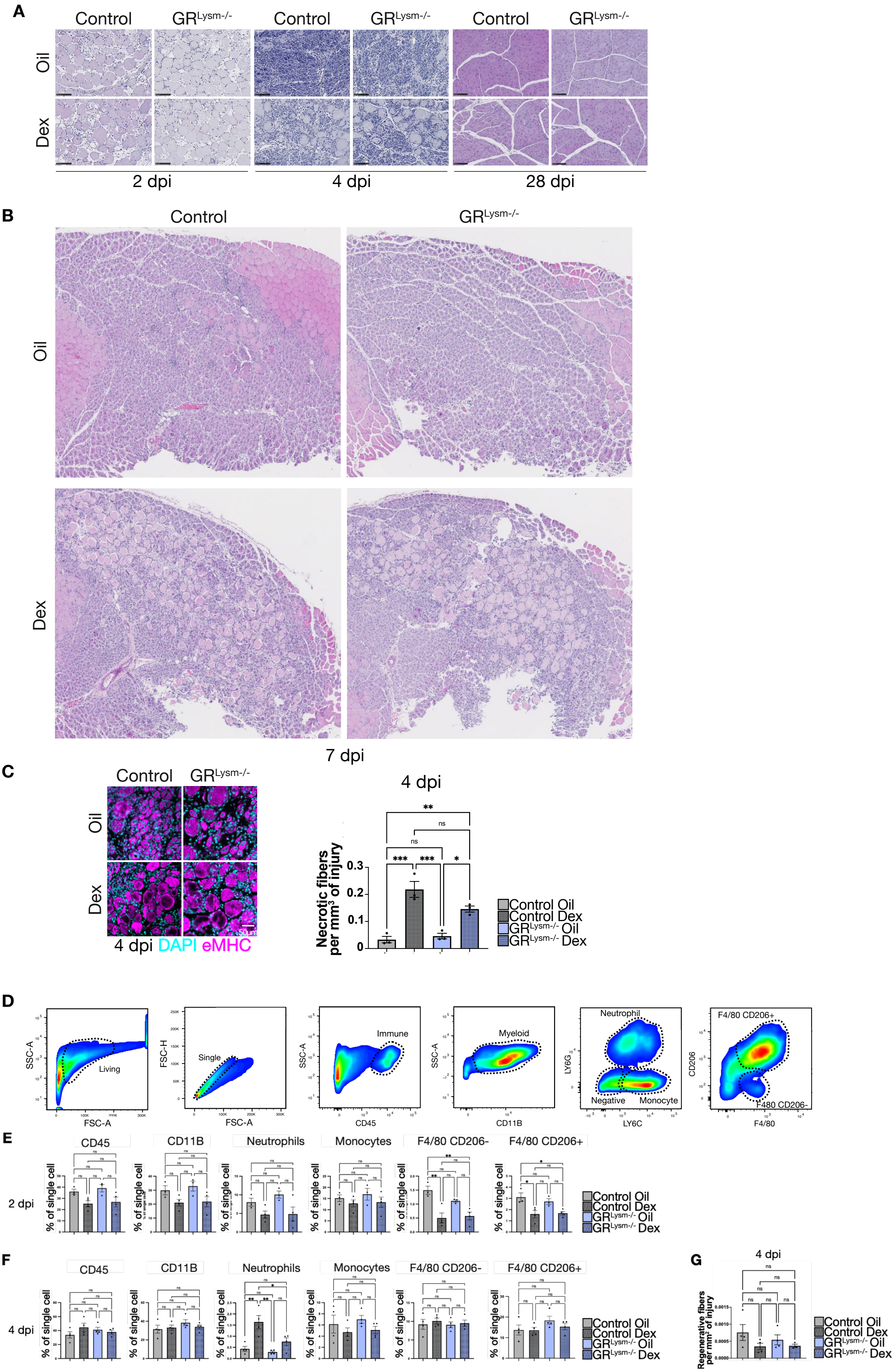

A
Control
GRLysm-/-
Oil
Dex
2 dpi
4 dpi
28 dpi
B
Control
GRLysm-/-
Oil
Dex
7 dpi
C
Control
GRLysm-/-
Oil
Dex
4 dpi DAPI eMHC
4 dpi
Necrotic fibers per mm3 of injury
Control Oil
Control Dex
GRLysm-/- Oil
GRLysm-/- Dex
D
SSC-A
FSC-A
Living
FSC-H
Single
Immune
CD45
Myeloid
CD11B
Neutrophil
LY6G
Negative
Monocyte
LY6C
F4/80 CD206+
CD206
F480 CD206-
F4/80
E
2 dpi
CD45
CD11B
Neutrophils
Monocytes
F4/80 CD206-
F4/80 CD206+
% of single cell
F
4 dpi
G
Regenerative fibers per mm3 of injury

Figure S4: Dexamethasone treatment during the pro-inflammatory phase impairs early muscle regeneration

(A-B) Representative (A) and full scan (B) hematoxylin and eosin staining of tibialis muscles (TA) of dexamethasone- (Dex) or vehicle-treated (Oil) control and $GR^{Lysm-/-}$ mice 2, 4 and 28 dpi (A) or 7 dpi (B). Scale bar, 100 µm.

(C) Representative immunofluorescent detection of eMHC (magenta) in tibialis muscles of Dex- or Oil-treated control and $GR^{Lysm-/-}$ mice 4 days after injury. Quantification represents the percentage of necrotic fibers relative to the injury surface. Control vehicle mice: n=3; $GR^{Lysm-/-}$ vehicle mice: n=3; Control Dex mice: n=3; $GR^{Lysm-/-}$ Dex mice: n=3. Statistical test used is Two-way ANOVA with Tukey *post-hoc* correction. The various comparisons were non-significant. Scale bar, 50 µm. Regenerating myofibers were identified based on eMHC positivity combined with morphological criteria, including central nuclei and fiber morphology. eMHC-positive necrotic fibers were excluded from quantification based on their distinct morphological appearance.

(D) Representative flow cytometry gating strategy used to identify myeloid populations in injured TA muscles. $CD45^+$ cells were first selected as immune cells, and $CD11b^+$ cells as myeloid cells. Neutrophils were defined as $Ly6G^+CD11b^+$ cells, monocytes as $Ly6C^+/Ly6G^-/CD11b^+$ cells, and macrophages as $Ly6G^-/Ly6C^-/CD11b^+/F4\text{-}80^+$ cells further subdivided into $CD206^-$ (M1-like) and $CD206^+$ (M2-like) subsets.

(E) Percentage of myeloid cells in 2-days-injured TA of Dex- or Oil-treated control and $GR^{Lysm-/}$. Control vehicle mice: n=3; $GR^{Lysm-/-}$ vehicle mice: n=3; Control Dex mice: n=4; $GR^{Lysm-/-}$ Dex mice: n=4. Data are presented as mean ± SEM. Statistical test used is Two-way ANOVA with Tukey *post-hoc* correction; ns, non-significant; * = $p < 0.05$; ** = $p < 0.01$; *** = $p < 0.001$.

(F) Percentage of myeloid cells in 4-days-injured TA of Dex- or Oil-treated control and $GR^{Lysm-/}$. Control vehicle mice: n=4; $GR^{Lysm-/-}$ vehicle mice: n=4; Control Dex mice: n=4; $GR^{Lysm-/-}$ Dex mice: n=4. Data are presented as mean ± SEM. Statistical test used is Two-way ANOVA with Tukey *post-hoc* correction; ns, non-significant; ** = $p < 0.01$; *** = $p < 0.001$.

(G) Quantification represents the percentage of regenerative fibers relative to the injury surface in 4-days-injured TA of Dex- or Oil-treated control and $GR^{Lysm-/-}$mice. Control vehicle mice: n=4; $GR^{Lysm-/-}$ vehicle mice: n=4; Control Dex mice: n=4; $GR^{Lysm-/-}$ Dex mice: n=3. Data are presented as mean ± SEM. Statistical test used is Two-way ANOVA with Tukey *post-hoc* correction; ns, non-significant; ** = $p < 0.01$; *** = $p < 0.001$

Supplementary Tables

Supplementary Table 1: Primers for mouse genotyping

Gene	Forward	Reverse
LysMCre	5’ TTCCCGCAGAACCTGAAGATGTTCG 3’	5’ GGGTGTTATAAGCAATCCCCAGAAATGC 3’
GRL2/WT	5’ AGATCATTTGCCTAGCAGGCATGAG 3’	5' GTCAACACATGATCACCTTGCAGTC 3'
GRL-	5’ CCAGAGAACTAATTGGCICTIGCAC 3’	5' GTCAACACATGATCACCTTGCAGTC 3'

Supplementary Table 2: Antibody list

Antibody / Dye	Reference
LY6C/G	Abcam AB2557
IBA1	Abcam AB5076
F4/80	Cell signaling 70076
KI67	R&D systems 14-5698-82
gH2AX	Sigma-Aldrich clone JBW301
WGA	W32464 thermofisher
MYOG	Abcam AB1835
PAX7	Thermo Fisher PA1-117,
ACTN1	Abcam ab9465
COL4	Sigma AB769
CD163	Abcam EPR19518, ab182422
CD45	Abcam AB10558
eMHC	Dshb F1.652
GR	Home made

Supplementary Table 3: List of GR partners identified by immunoprecipitation followed by mass-spectrometry

See Table S3.xlsx
